# Use and perception of risk: traditional medicines of Pakistani immigrants in Norway

**DOI:** 10.1186/s12906-024-04620-0

**Published:** 2024-09-07

**Authors:** Saliha Khalid, Agnete Egilsdatter Kristoffersen, Lise-Merete Alpers, Christine Råheim Borge, Samera Azeem Qureshi, Trine Stub

**Affiliations:** 1 The National Research Center in Complementary and Alternative Medicine (NAFKAM), Department of Community Medicine, Faculty of Health Sciences, UiT The Arctic University of Norway, Tromsø, N-9037 Norway; 2 VID Specialized University, Postboks 184, Oslo, 0319 Norway; 3 Lovisenberg Diaconal Hospital, Oslo, Norway; 4 Norwegian Institute of Public Health, Oslo, Norway

**Keywords:** Health practices, Herbalism, Medical pluralism, Pakistani immigrants, Traditional medicine

## Abstract

**Background:**

Pakistani immigrants are the largest non-Western ethnic minority group in Norway. Traditional medicines (TM) are extensively used in Pakistan, and studies show that ethnic minorities also use them to recover from illness after migration to the Western world. This study aims to explore Pakistani immigrants’ experiences and perceptions of risk regarding the use of TM to treat illnesses.

**Methods:**

A qualitative study was conducted through in-depth interviews (*n* = 24) with Pakistani immigrants in Norway from February to March 2023. Participants were recruited through purposive and snowball sampling methods. The data was analyzed using Braun & Clarke’s reflexive thematic analysis (RTA) using Nvivo.

**Results:**

RTA revealed three main themes and six sub-themes. The main themes were: (a) House of knowledge, (b) Choosing the best possible approach for health restoration, and (c) Adverse effects of TM used. A total of 96 different TM were identified, including herbs, food items, animal products, minerals, herbal products, and ritual remedies. All participants used TM to restore health in acute and chronic diseases, and many used TM along with conventional medicines. The participants’ mothers were the primary source of knowledge about TM, and they passed it on to the next generation. They also frequently used religious knowledge to recover from illness. Although TM is considered safe because of its natural origin, some participants experienced adverse effects of TM, but none of them reported it to the health authorities.

**Conclusion:**

The study helps to understand the experiences and perceptions of risk of Pakistani immigrants in Norway regarding traditional practices for treating health complaints. Public health policies to improve the health of these immigrants should consider the importance of TM in their lives. Further research is necessary to explore the safety and toxicity of those TM that are common in Pakistani households in Norway.

**Supplementary Information:**

The online version contains supplementary material available at 10.1186/s12906-024-04620-0.

## Background

Migration has affected the Western world in various aspects, but its contribution to several healthcare challenges is a significant consequence [1]. The diverse cultural beliefs of the patients can hinder healthcare providers from holistically addressing the health complaints of the immigrant community [2]. As Norway has experienced substantial growth in immigration in recent decades, immigrants and their Norwegian-born children accounted for 20.8% of the population in 2024 [3]. One of the largest non-Western ethnic minority groups in Norway is the Pakistanis, with 23,569 immigrants and 18,662 born to immigrant parents in 2024 [4]. Most live in the capital city of Norway, Oslo, and the areas around it [4]. More than 50% of the Pakistani immigrants in Oslo report poor health as opposed to only one-fifth of ethnic Norwegians [5]. This is partly due to a higher rate of diabetes (14% vs. 2.6%) [5], psychological distress (22% vs. 9.9%) [5], and cardiovascular disease (acute myocardial infarction, 5.7% vs. 2.7%) [6]. It leads to more frequent general practitioners and medical specialists visits than ethnic Norwegians [7]. This cultural and ethnic diversity among healthcare seekers comes with variations in disease conditions [5, 8], healthcare expectations [9], and patients’ beliefs [10]. Different ethnic groups use traditional medicine (TM) to manage illnesses in Norway [11, 12]. TM is extensively used by more than half of the population in Pakistan [13, 14], and research has demonstrated that Pakistanis continue using TM after emigration from Pakistan [1, 15]. Therefore, knowledge about their traditional practices is needed to meet immigration-related healthcare challenges, as we know little about them [16].

In Pakistan, both private and public healthcare facilities are available [17]. The public sector is underutilized due to unequal resources in the population, lack of trained staff, lack of access, poor governance, etc. [18–21]. In this context and due to financial shortcomings, self-medication, self-care, herbal medicines, and TM are used by more than half of the population to restore health [13, 14, 22]. TM is known as *“the knowledge*,* skills*,* and practices based on the theories*,* beliefs*,* and experiences indigenous to different cultures*,* whether explicable or not*,* used in the maintenance of health as well as in the prevention*,* diagnosis*,* improvement*,* or treatment of physical and mental illness”* [23]. In Pakistan, TM is a part of the cultural heritage and is preferred by 51.7% of the Pakistani population today, of which 20% combine it with conventional medicine [24, 25]. Tibb-e-Unani, homeopathy, mind-body medicine, and biologically based practices (home remedies, diet, and nutrition) are commonly used in Pakistan [24, 26] for colds, coughs, gastrointestinal problems, etc [27].

As treating illnesses with TM and their self-administration is deeply embedded in Pakistani cultural heritage [28], research indicates that Pakistanis maintain the use of TM even after emigrating from Pakistan. Pakistani immigrants in England were found to associate certain foods with medicinal effects [29]. Other international studies show that Pakistani immigrants still use TM modalities in their new home country [1, 15, 30]. So, Pakistani immigrants may also widely use TM in Norway. Medical pluralism is a concept that explains why individuals resort to different modalities and treatments to pursue health in any community [31]. This leads to using conventional medicines and TM, even when these have mutually incompatible explanations for the illness [32].

One of the goals of the World Health Organization (WHO) Traditional Medicine Strategy 2014–2023 is to support the safe use of TM. Considering this, asking TM users about their perceptions of risk and experiences regarding such use is essential. Risks associated with healthcare intervention may be categorized as direct or indirect risks [33]. Direct risk includes harm from a medical intervention, procedure, product, or treatment, including negative interactions between conventional medicines and TM interventions like herbs and supplements. Direct risk is any adverse effects from using any herb, food item, supplement, or health-related practice [34]. Indirect risk is not directly related to a specific medicine or herb but harms the patient due to the treatment setting or practice in general [35], such as untrained health professionals, poor communication between healthcare professionals and patients, lack of authentic information, etc [33, 35]. According to a systematic review of adverse drug reactions (ADR) in Norway [36], 250 out of 260 ADR reports of plant-based products submitted to RELIS (Manufacturer-independent drug information for healthcare professionals) were related to herbal dietary supplements [37]. Therefore, it is essential to consider the safety of herbal products before they are used and to record the experience-based knowledge of TM users about the safety of these TM.

Even though Hakonsen et al. found that 15% of first-generation Pakistani immigrants in Norway used herbal remedies and supplements bought or sent from Pakistan [16], we do not know what herbal remedies they use, why and how they use them, and their beliefs about such use. This knowledge about cultural beliefs and traditional practices can help healthcare providers better understand this patient community and address their health complaints holistically [2]. This better understanding can improve communication between immigrants and healthcare providers, finally leading to patient trust in the healthcare system [38]. This knowledge is also essential for patient safety and to avoid negative interactions between natural remedies and conventional medicines [39].

So, in this study, we will explore Pakistani immigrants’ experiences with using TM to treat illnesses. Emphasis was placed on exploring various modalities, such as food items, herbal remedies, and dietary supplements. Furthermore, we intended to delineate the perceived safety profile of these TM practices from the participants’ perspective (perceptions), thereby contributing to a nuanced understanding of the intersection between cultural practices and health within this community.

## Methods

We used in-depth interviews to explore Pakistani immigrants’ experiences regarding TM use in Norway from February to March 2023. A qualitative research design helps understand the phenomena of sparse knowledge [40] and further generates a deep understanding of the informant’s knowledge, experiences, attitudes, and feelings [41]. In this study, we have used the COnsolidated criteria for REporting Qualitative research (COREQ) checklist [42].

### Study area and setting

**Fig. 1:**
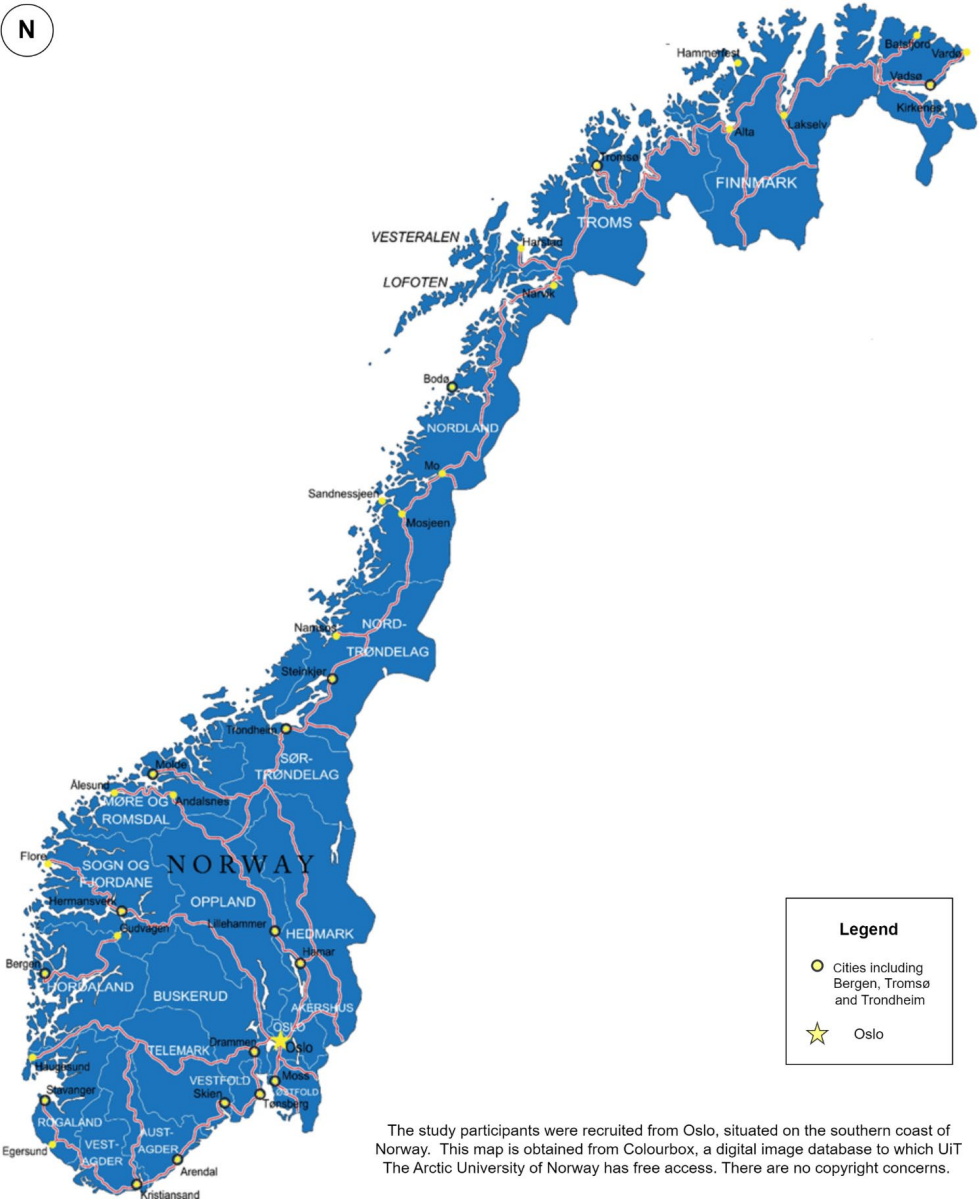
Map of Norway

The study took place in Oslo, the capital of Norway, which is known for its significant investment in public health. Oslo is the most populated city in Norway, located on the country’s southern coast. Figure 1 shows Oslo’s location in Norway. In 2024, 34.7% of the population in Oslo corresponds to immigrants and their children, and 13.6% of them are from Asia [3]. Norway allocates substantial resources to healthcare, corresponding to 12.7% of mainland Norway’s Gross Domestic Product in 2022 [43]. The Norwegian healthcare system is predominantly financed by public funds and the government, which warrants low personal financing [44]. Conventional medicine is Norway’s officially approved medical system [45, 46]. However, Traditional and Complementary Medicine (T&CM) is practiced outside the official healthcare system and is used by 62% of the population annually [47].

### Recruitment

Participants were recruited from patients in the outpatient department of a local hospital setting and their community. The inclusion criteria for the study participants were: (1) First-generation (born in Pakistan) or second-generation (born in Norway with both parents born in Pakistan) immigrants from Pakistan living in Norway, (2) first-hand experience of using TM, (3) ability to understand Urdu, Norwegian, or English, (4) For those recruited via hospital, the reason for admission to the hospital was not intoxication for suicidal purposes. The exclusion criteria were age under 18 years, mental impairment, and hospital inpatients. Purposive sampling [40] was used to recruit participants who visited the hospital’s medical unit (*n* = 6). An introduction letter describing the study was distributed to the hospital staff so that they could inform the patients about the study. The hospital staff also provided the patients with written information about the study in a patient information brochure (PIB). PIB was available in Norwegian, Urdu, and English. When a patient agreed to receive more information about the study, the first author received their contact information. She provided more in-depth information through a telephone call, including the reasons for doing the research, and scheduled a time for a personal interview. Six participants, all first-generation immigrants, were recruited through this method. These participants helped recruit other participants (*n* = 18) by snowball sampling [40]. The interviewer had no prior relationships with the study participants. One of the immigrants refused to participate in the study due to lack of time, but none of the participants dropped out.

### Advisory group

A group of 5 voluntary individuals (1 male and four females), including first- and second-generation immigrants, contributed to ensure public involvement in the project. The group gave valuable feedback on the interview guide, patient information brochure’s content, design, and graphics; and supported translating the brochure into Urdu. This group was not involved in analyzing the data and drafting the manuscript. The members were not paid for this service and were not participants in the study.

### Interview guide and training of the interviewer

An interview guide was developed based on existing literature with the help of experienced qualitative researchers in the research group, including TS, LMA, and SAQ, a female Pakistani immigrant researcher in Norway. After two pilot interviews, the interview guide was shortened. No changes have been made to the interview guide between the interviews.

The first author (SK) is a PhD student and a pharmacist from Pakistan. She was brought up in Pakistan and knows about Pakistani culture. AEK and TS listened to the two initial interview recordings in English. SAQ (researcher and co-author) participated in one of the face-to-face interviews in Urdu with SK. AEK, TS, and SAQ provided feedback about the interviewing method and follow-up probing questions. It helped to ensure reflexivity in the study, a process of critical reflection on the self as a researcher [40, 48]. It helped SK to identify her blind spots where she could influence the interview. This input made her more aware of her position as a researcher and ensured that the information she collected aligned with the purpose of the study. (The interview guide is available as supplementary material)

### Data collection

Twenty-four in-depth interviews lasted between 30 and 90 min and were conducted by the first author either face-to-face (*n* = 18) or online (audio) (*n* = 6). Although face-to-face communication may be more effective due to expressions and gestures, we gathered in-depth information in the online interviews due to cultural similarity and lack of language barrier between the participants and the interviewee. All the interviews were audio-recorded after the participant’s consent. The face-to-face interviews were conducted at the place of the participant’s choice: In a hotel (*n* = 3), library (*n* = 5), and private homes (*n* = 10) from February to March 2023 in Oslo. Seven out of 24 interviews were conducted in English, while the others were in Urdu. Two members of the research team, who are native Urdu speakers and fluent in English, contributed to the translation process. The first author translated and transcribed the interviews verbatim into English and read the transcripts several times to minimize errors. The translations were cross-checked by another team member and finalized after gaining a consensus. Data saturation was reached at 22 interviews, and two more interviews were conducted afterward to ensure we didn’t obtain any new information [40]. The first author took notes during the interviews. No repeated interviews were carried out.

### Data analysis

Following the reflexive thematic analysis (RTA) method by Braun and Clarke, two authors (SK and TS) analyzed the interview transcripts [49, 50]. RTA is a six-phase method including (1) familiarising with the data, (2) generating codes, (3) constructing themes, (4) reviewing potential themes, (5) defining and naming themes, and (6) producing the report [49, 50]. SK and TS familiarised themselves with the data by reading and re-reading the transcripts and marking features of interest. Twenty-five codes were developed through an inductive orientation, which later merged into seven themes. These themes were defined and named multiple times before finalizing them. After discussions with the research team, themes with similar information were collapsed into three main themes. Six sub-themes were created to show diversity in the data. For example, the sub-theme, “Exchange of health practices between Pakistan and Norway,” was originally a theme named “exchange between different regions of the world” with two sub-themes: (1) Exchange of knowledge and (2) Exchange of entities. Later, we named it cross-cultural exchange, then cross-medical exchange, and finally, “Exchange of health practices between Pakistan and Norway.” At the end of the analysis, this theme was made a sub-theme under the theme, “Choosing the best possible approach for health restoration,” as it was the primary reason for this exchange. All the analysis steps in RTA were done iteratively, and this reflexive approach helped the investigators understand the data and name the themes better than in a linear process. To ensure the research results truly reflect reality (internal validity, credibility, and study triangulation), both SK and TS performed the data analysis [48], and the consolidated criteria for reporting qualitative studies were used [42]. Analysis was conducted using QSR-NVivo v10.0 software [51].

### Ethical considerations

The study was approved by the Norwegian Centre for Research Data (reference number: 447080) as the Regional Committees for Medical and Health Research Ethics (REK 493745) decided that the study was not considered health research in Norway and, therefore, did not require approval from them. The participants were informed about the study’s purpose, method, and content and their rights as participants. It was emphasized that participation was voluntary and that they could withdraw without consequences. Verbal and written informed consent were obtained from the participants before recording the interview. Each participant received an identification (ID) number to ensure anonymity. The confidentiality of the participants was ensured by anonymizing the interview transcripts. We didn’t return the transcripts to the participants for comments/corrections, but one of the participants reviewed her transcript upon request and did not suggest any changes.

## Results

### Demographics of the participants

A total of twenty-four participants (sixteen females and eight males) ranging from 21 to 80 years of age were interviewed (Table 1). Sixteen participants were first-generation Pakistani immigrants, and eight were second-generation immigrants. More than half of the participants were females. The mean age of 1st generation participants was 50 years and 29 years for the 2nd generation participants, respectively. All the 1st generation participants were married. Two 2nd generation participants were married, and others were single (*n* = 6). All participants were living with their families in Norway. Less than half of the participants were retired. Others were self-employed or students. Diabetes (*n* = 5), digestive problems (*n* = 8), and cardiovascular problems (*n* = 6) were the most frequently reported health complaints by the participants. The number of different TM modalities used by the participants ranged from 6 to 27, with a mean of 14 different TM modalities per participant. Table 1 shows the participants’ demographic data and the types of TM modalities they used.

**Table 1:** Demographic data of the participants

ID #	Gender	Age* in years	Years since migration (mean = 25.5 years)	Number and types of TM modalities used
F1	Male	41–50	31–40	6 (food, herbs, ritual remedies, herbal products)
F2	Female	61–70	11–20	23 (food, herbs, minerals, animal products, ritual remedies, herbal products)
F3	Male	71–80	51–60	6 (food, herbs, animal products)
F4	Male	41–50	41–50	12 (food, herbs, minerals, animal products, herbal products)
F5	Male	71–80	51–60	10 (food, herbs, minerals, animal products, herbal products)
F6	Female	41–50	21–30	18 (food, herbs, animal products, herbal products)
F7	Male	61–70	31–40	14 (food, herbs, minerals, animal products, herbal products)
F8	Female	31–40	1–10	27 (food, herbs, minerals, animal products, herbal products)
F9	Female	61–70	41–50	22 (food, herbs, animal products, herbal products, ritual remedies)
F10	Female	61–70	41–50	11 (food, herbs, minerals, animal products)
F11	Female	61–70	41–50	6 (food, herbs, animal products)
F12	Female	31–40	1–10	11 (food, herbs, animal products, herbal products)
F13	Female	31–40	1–10	8 (food, herbs, animal products, herbal products)
F14	Female	31–40	1–10	15 (food, herbs, minerals, animal products, herbal products, ritual remedies)
F15	Female	21–30	1–10	15 (food, herbs, minerals, animal products)
F16	Male	21–30	1–10	13 (food, herbs, minerals, animal products, herbal products)
F17	Female	21–30	Born in Norway	18 (food, herbs, minerals, animal products, herbal products)
F18	Female	21–30	Born in Norway	22 (food, herbs, minerals, animal products, herbal products)
F19	Male	21–30	Born in Norway	7 (food, herbs, herbal products)
F20	Female	41–50	Born in Norway	11 (food, herbs, animal products, herbal products)
F21	Female	41–50	Born in Norway	17 (food, herbs, minerals, ritual remedies, animal products, herbal products)
F22	Male	21–30	Born in Norway	9 (food, herbs, minerals, herbal products)
F23	Female	21–30	Born in Norway	21 (food, herbs, minerals, animal products)
F24	Female	21–30	Born in Norway	14 (food, herbs, minerals, herbal products, ritual remedies)

*For anonymization purposes, the age is reported within an age range of 9 years

### Themes

The analysis of the interviews revealed three main themes: (a) *House of Knowledge*, (b) *Choosing the best possible approach for health restoration*, *and* (c) *Adverse effects of TM use*. We divided these themes into six sub-themes (see Table 2 for the Overview of themes and sub-themes). We did not recognize any pattern of differences between the views of 1st and 2nd generation immigrants.

**Table 2:** Overview of themes and subthemes

Themes	Subthemes
a. House of Knowledge	- Influence of family in TM use - Knowledge about TM for specific illnesses
b. Choosing the best possible approach for health restoration	- Health-seeking behavior for restoring health - Reasons for using TM - Exchange of health practices between Pakistan and Norway
c. Adverse effects of TM used	- Participants believe that natural is always safe

#### House of knowledge

Under this theme, we explained the participants’ extensive knowledge of TM for specific illnesses. We gained insight into the use of TM while living in a family system and how family influenced TM use. We explored the importance and role of family in sickness and cure.

##### Influence of family in TM use

Most participants talked about the importance of family in the lives of immigrants when they were sick and how the family influenced their TM use. All the participants lived with their families in Norway and shared that the source of knowledge regarding TM is their home (mothers and grandmothers). Some of the participants expressed that the Pakistani family system influenced their lives in Norway. For instance, one participant (F16) explained it this way: *The family system is very strong in Pakistan. If you live in a family*,* you learn these things automatically. They used to apply these things [TM] to you and your siblings*,* and their parents did the same with them. So*,* when you live in such a society*,* you learn these things from your family life.* This quote explains how living with and observing the family helped the participants learn about TM. The participants’ belief in the healing power of TM and these learned behaviors reinforced the notion that TM could benefit their health because of living with the family. A second-generation participant (F23) who was a frequent user of TM with various health complaints stated: *It is because of the environment we have at home. I was also always observing my parents*,* and they just took Paracetamol as a last resort. They always tried to recover their body using natural things.* This explains how the family environment transferred one generation’s beliefs regarding TM to another.

There were also participants who mentioned that discussing with family members is essential when they experience mental or physical health complaints and deciding on TM to be used. A participant (F3) stated: *As a family first*,* I take suggestions from my wife that I have this problem and ask what I should do now. So*,* I take her suggestions; whatever she recommends*,* like some medicine or any remedy*,* herb*,* kawa*,* then I take it.* Another participant (F1) who received family care in the past stated: *My family helped me so much during this time [with schizophrenia]. Everyone took great care of me at home and behaved very well with me.* So, it shows how living with the family and family care provided physical, emotional, and mental support in illness. Some participants also stated that they always contacted their mothers when they experienced a health complaint because they were sure their mothers would suggest a remedy for every problem. F17 explained: *I call my mother*,* and then*,* of course*,* she would bring out a remedy.* So, trust in the family and receiving their help and solutions seemed important, even for adults. It shows the connection with the family, family care, and dependence on the traditional knowledge of the family to recover from the health complaints.

##### Knowledge about TM for specific illnesses

Participants shared their extensive knowledge about TM and how they use it for specific illnesses. The information they shared included common names of TM, preparation methods, dosage, adverse effects, and lifestyle advice. We identified 96 different TM modalities used for 44 different health complaints. Most reported TM modalities were medicinal plants, animal products, and food items, but the participants also reported using some minerals, ritual remedies, and commercial herbal preparations (Table 2). Generally, the women and participants with chronic illnesses used more TM modalities than the men and other participants. Table 3 shows TM used for various health complaints and the participant-reported adverse effects of TM. An extended list with more information about TM, including traditional names, English names, scientific names, preparation methods, dosage, adverse effects, and lifestyle advice, is available as supplementary material (additional file 1: Table 4).

**Table 3:** TM used for specific health complaints and participant-reported adverse effects of TM used

Health complaint	Traditional medicines used	Adverse effects experienced from TM use
Alopecia	Garlic, onions, cumin seeds	-
Arthritis	Turmeric, sesame seeds, fenugreek seeds	-
Allergy	Black salt. Soak the food items to which you are allergic in water overnight, peel them, and eat them in the morning, e.g., almonds	-
Asthma	Turmeric, fennel seeds, cloves, honey, green cardamom, peppermint, salt, khamira	-
Backache	Turmeric, raw egg, milk, fox nut, almonds, walnuts, cashew nuts, pistachio, sunflower seeds, flaxseeds, pumpkin seeds, sesame seeds, fennel seeds, and jaggery	-
Bloating	Carmina, hajmola, colic drops, castor oil	-
Body pain	Turmeric, milk, honey, warm water, mustard oil	-
Bronchitis	Khamira, joshanda	-
Cardiac problems	Peppermint, garlic, ginger, olive oil, rapeseed oil, and clarified butter. Use peppermint leaves in the salad	-
Cold + flu	Joshanda, warm water, honey, turmeric, clove, cinnamon, ginger, green cardamom, green tea, chicken and sweetcorn soup, star anise, black cardamom, neem leaves, carom seeds, Vicks, boiled eggs, chicken broth, garlic, white salt, black pepper, peppermint, on-guard oil, lemon, milk, black tea	Overuse of honey causes stomach-burning
Constipation	Barley, plums, raisins, warm water, black salt, psyllium husk, yoghurt, fennel seeds, green cardamom, green tea, carom seeds, malt extract, and figs. For cramps and constipation, increase daily water intake	-
Cough	Joshanda, cinnamon, star anise, black cardamom, honey, dry ginger, clarified butter, salt, black pepper, nutmeg, mace, fennel seeds, green cardamom, peppermint, ginger, turmeric, mulberries, lal-sherbet, cloves, eggs	Excessive use of ginger can cause bloating
Diabetes	Bitter gourd, neem leaves, black cumin (cures anything except death), fenugreek seeds, cinnamon, peppermint, fennel seeds, green cardamom, guava leaves, Krom and black plum	Java plum causes a bad smell
Diarrhea	Cinnamon, peppermint, fennel seeds, green cardamom, rice water, banana, yoghurt, psyllium husk, peel of pomegranate, carom seeds, black salt, black cardamom, lemon	-
Eczema	Lavender oil (always dilute before use)	Causes skin burn when not diluted
Eye infection	Saltwater	
Fever	Water	
Hair loss	Coconut oil, eggs, onions, cumin seeds, almonds, rosemary oil, vitamin E oil, castor oil	Bad odor of oils
Headache	Black tea, green cardamom, salt	
Heartburn + indigestion	Peppermint, fennel seeds, green cardamom, carom seeds, ginger, black cumin seeds, psyllium husk, green tea, cinnamon, green chilies, Carmina, yogurt, milk, black salt, Norwegian herbal tea, chirata leaves	Chirata leaves are very bitter
Hyperlipidemia	Rapeseed oil and cinnamon	-
Hypertension	Black cardamom, peppermint, psyllium husk, basil seeds, honey, black carom seeds, dried apricots	-
Irritable bowel syndrome	Psyllium husk, olive oil, peppermint, green cardamom, fennel seeds, dates, figs, pomegranate, honey, black cumin seeds	-
Iron deficiency	Pomegranate, meat, spinach	-
Kidney disease	Vinegar (reduces creatinine level)	-
Hypotension	Cloves, seeds of green cardamom, boiled egg, salt	-
Menstrual pain	Clarified butter and warm milk. Avoid cold-effect food like lassi/milkshakes	-
Migraine	Dry ginger + white rice	-
Miscarriage	Use fish, fruits, vegetables, and vitamins. Reading a chapter of the Quran and wearing an amulet	-
Mosquito bites	Mospel, boric powder	-
Multiple sclerosis	Peppermint oil, frankincense oil, on-guard oil. Massage specific points beneath the feet with these oils if you have a headache	-
Oral care	Alum, fennel seeds, rock sugar	-
Peptic ulcer	Goji berries, dried mangoes, dried cranberries. All nuts are good	-
Prolapse	Do exercises	-
Schizoprenia	Reading darood sharif (a supplication)	-
Skin burn	Potato sucks the warm part from the skin	-
Skincare	Lemon, avocado, cucumber, aloe vera. Make masks from them and use them	-
Skin scratch	Sulfur water. Take a bath in a sulfur pond in Pakistan	-
Sore throat	Cloves, cinnamon, turmeric, mouthwash, joshanda, honey, black pepper, warm water, salt water, Wheat, green cardamom, fennel seeds, ginger, neem tree stem + clarified butter, wood ash, guava leaves, black cumin, mustard oil, garlic, Vicks, dox, salt, peppermint, rose petals	Salt in the throat causes pain, nausea, and bleeding.
Toothache	Cloves, mouthwash, white salt	-
Urinary infection	Turmeric, cranberries, honey, and lemon	Bad taste of turmeric
Vomiting	Apple, peppermint, green cardamom	-
Obesity	Fasting, rocket leaves, cinnamon, honey, lemon, psyllium husk	-
Wound/infection	Alum, turmeric, olive oil, garlic, ginger, onions	-

#### Choosing the best possible approach for health restoration

Throughout this theme, the participants described how they chose the best possible option for restoring health while living in Norway and what factors govern this decision. They explained how living in Norway influenced their attitudes and behaviors toward health-seeking. Three subthemes were identified under this theme: health-seeking behavior for restoring health, reasons for using TM, and the exchange of health practices between Pakistan and Norway.

##### Health-seeking behavior for restoring health

We identified different health-seeking behaviors of the participants for restoring health in illness and their beliefs behind these behaviors. Many of the participants reported using both TM and conventional medicines to recover from health complaints. The decision about which modalities to use could depend on factors such as their belief in TM, the nature and severity of the illness, access to the healthcare system, the response from the healthcare providers, and the availability of TM at home. They also expressed that using TM was their first approach to any health complaint. However, two of the participants (F23, F20) stated that the approach they used to manage the illness depended on their problem. In addition, a couple of participants (F1, F13) mentioned that their first approach was using conventional medicines. Some participants suffering from chronic diseases expressed the importance of taking conventional medicines to maintain their quality of life.

A few participants preferred to use TM for minor illnesses but usually visited the conventional doctor in case of serious diseases. Various reasons were identified for this health-seeking behavior. Some participants believed that TM could cure the problem entirely with few adverse effects rather than eliminate the symptoms. F19 explained that *keeping it natural positively helps your body because painkillers can have negative effects. What it [painkillers] can do is that the problem will be there*,* but your pain will not be there. But the problem can still be there*,* if you know what I mean*. So, it depicts a strong belief in the healing power of TM. Some participants (F20, F21) used TM because of the Islamic faith that Allah created natural things that are better to use. It included the fact that the food items specified in the Quran and Hadees (teachings of the prophet) can cure the illness. A female respondent (F21) said: *It’s more related to my belief in Islam*,* and I want to use the natural things which God gave us instead of all these chemically produced things [conventional medicine].*

Another reason for using TM was the belief that the long-term use of conventional medicines is not suitable for the body. One of the participants (F14) managed her problem of chronic constipation through diet changes and household remedies because she found it better to avoid conventional medicine prescribed by her doctor. The difficulty in assessing the healthcare services in Norway was another reason for the preference for TM in illness. A female informant (F2) said: *I have a lot of these things [TM] at home because here [in Norway]*,* it is very difficult to reach the doctor when you are sick. As they have a very long procedure*,* first you have to talk to the doctor*,* and then he will give you an appointment.* Others preferred TM due to the low probability of getting conventional medicines from the healthcare system compared to Pakistan. All the participants who had children under five years of age reported frequent use of TM for their children, and they believed that it helped them. A worried mother (F15) of three small children who frequently suffered from seasonal allergies and infections stated: *The doctor just gave her [child] paracetamol because there is no other medicine like cough syrup for a child in Norway who is under two years of age. Apart from paracetamol*,* the remedy I use is adding black pepper powder or nutmeg to honey and mace powder to honey and giving it to her.* These quotes depict that barriers in assessing the Norwegian healthcare services contributed to using TM for themselves and their families.

Some participants (F13, F1) also preferred to use conventional medicines as a first choice, even for minor health complaints, as they wanted quick relief. In addition, some participants reported using conventional over-the-counter medications before accessing the Norwegian healthcare system and in the waiting time to see the doctor. F15 stated: *I always want a quick solution. I always start with paracetamol for me and my daughter. I don’t visit the doctor immediately when the problem starts.* The participants (F16, F5) revealed they were using more conventional medicines after migration because of the lack of TM in Norway. Regarding this, the participant (F16) stated: *In Norway*,* it is difficult to get those things from which we do the remedies in Pakistan. So*,* I didn’t do anything.* In short, although TM was the first choice to treat illness for most participants, several combined this with conventional medicine or entirely used conventional medicines due to the lack of availability of TM in Norway.

##### Reasons for using TM

We identified that the participants used TM often in combination with conventional medicine to manage acute and mild health complaints, chronic problems, adverse effects of conventional medicines, and undiagnosed health complaints. Participants reported using different herbal remedies, food items, and commercial herbal preparations for acute and mild health complaints such as flu, seasonal allergies, and gastrointestinal problems. Several participants (F15, F4, F23, F8, F14) mentioned that they preferred TM for minor complaints because the doctors in Norway usually don’t prescribe any medication for minor complaints (cold and viral infections).

The participants (F4, F6, F7, F2, F3) also expressed that they combined TM with conventional medicine for chronic conditions such as diabetes, arthritis, and cardiovascular problems. For instance (F2): *Sometimes it happens*,* and you are taking medicine*,* but your sugar level is still not under control. Then*,* in this case*,* I use black cumin and fenugreek seeds*,* mix them in the water*,* and drink them. It makes the sugar level better*. Another participant (F6) mentioned using TM to control her blood pressure after taking conventional medicines. She stated: *I used to drink cold drinks after adding ice and psyllium husk to them. It lowers my blood pressure*,* and I also keep the seeds of black cardamom in my mouth. It is very effective.*

Some participants (F10, F11, F4, F3) reported using TM to manage chronic problems after experiencing adverse effects of conventional medicines. A participant (F10) had gastric problems due to extensive use of conventional medicines, so she started using TM (turmeric and seeds drinks). She (F10) found that TM could improve her quality of life: *That [turmeric drink] is so soothing*,* and I sleep like a baby*,* and I wake up without crying like a baby because it relieves my pain*. Another participant (F11) mentioned that she didn’t use conventional medicines because they make her drowsy, while another one (F4) avoided them to sleep peacefully.

Two participants used TM to manage autoimmune diseases because they knew that conventional medicines couldn’t cure their problem, so they preferred to use various home remedies and ritual remedies to manage their condition. F5 reported using TM to treat stomach problems that his doctor was unable to diagnose after multiple medical tests. He stated: *I had very severe burning in my stomach*,* and they (doctors) always said that everything was fine. But then my sister told me to add psyllium husk to milk at night and to drink it in the morning. I used it for just one week*,* and I don’t have any problem now after 30 years*. These data demonstrate the extensive dependence of first and second-generation participants on TM use in various types of health complaints.

##### Exchange of health practices between Pakistan and Norway

Participants reported the exchange of TM, conventional medicines, food items, and knowledge regarding health and TM between Pakistan and Norway. Some participants developed medical practices that were a mix of traditional and conventional medical practices. It was also expressed that they learned how to take care of their health through diet, exercise, and hygiene while living in Norway, and they continued practicing this when they visited Pakistan. For instance, a participant (F19) avoided eating in restaurants in Pakistan because of hygiene problems. Another participant (F21) continued eating in restaurants in Pakistan after eating raw red onion (quercetin: anti-bacterial) before eating anything else. She reported that she never got sick from food after she started this practice. It shows how the participants practiced their new (Norwegian) and traditional (Pakistani) knowledge of health for their well-being.

Participants (F8, F15, S2, F6, F3, F24) contacted herbalists and traditional healers in Pakistan while living in Norway through some family member or when visiting Pakistan due to family recommendations and the belief in TM. Some participants searched for remedies on the internet when they experienced some health complaints (F10, F14, F2, F24). Participants also reported bringing TM from Pakistan, Sweden (F4), and Turkey (F5, S2) because they were not available in Norway. Others took conventional medicines (F9, F10) and food items from Norway (F7) to Pakistan, doubting the quality of medicines in Pakistan. Some participants (F10, F1) reported that the medicines available in Pakistan are not “*first-class medicines*.” A participant (F1) with chronic illness explained it: *It means true and false. In Pakistan*,* false medicines (author’s comment: low quality) are very common and have fewer effects*. A participant (F7) who has been living in Norway for 35 years and has a gluten allergy always brings gluten-free flour to Pakistan as he can’t eat the flour available in Pakistan. These data depict how the exchange of TM and health practices between Pakistan and Norway occurred at different levels and contributed to practicing the best approach to health restoration.

#### Adverse effects of TM use

Under this theme, we explored the participants’ knowledge, experiences, and beliefs regarding the adverse effects of TM. Most of the participants were unaware of the adverse effects of TM and seemed confident that it could never happen. Few participants (F14, F21, F22) experienced minor adverse effects from honey, ginger, salt, and turmeric, such as an unpleasant taste or odor, bloating, and irritation (Table 3). However, one participant (F1) reported serious adverse effects. He experienced renal failure because of using TM recommended by a healer in Pakistan. He said that a religious healer advised him to eat sugar. He continued: *It harmed me significantly*,* so I was close to the death point many times*. He also reported a negative interaction of TM with conventional medicines in this way. *It happened that they (TM) lowered my sugar level so much. My doctor told me this is the problem with these remedies and that other medicines also stopped working after using them.* It depicts participants’ experiences regarding the adverse effects of TM and drug-herb interaction.

After experiencing adverse effects of TM, some participants (F1, F14) stopped using them, while others continued using the TM after changing their method of use and preparation. F14 stopped using honey and lemon because of the warm effect of honey on the body. She stated: *By a warm effect*,* I mean that you feel a burning in your stomach*. A participant (F21) had skin burns because of using concentrated lavender oil. She continued using it after mixing it with another oil. Two participants (F10, F4) reported allergic reactions (sore throat, breathing problems, and watery eyes) after eating almonds and figs. They continued using them after soaking the almonds and figs in water overnight and eating small portions of such food items as recommended by their healers. It led to no allergic reactions, according to the participants. It shows that participants had their ways of managing adverse effects, and many participants who experienced adverse effects continued using them despite experiencing these harms.

Some participants (F16, F12) were concerned about the adverse effects of TM before use. They used Google to search the pros and cons of TM, but none reported using scientific sources for this purpose. One participant (F12), the mother of two children, mentioned that she used to search on Google for remedies that were considered safe for her children. She believed that because of this counterchecking, her children never experienced any adverse effects of TM.

##### Participants believe that natural is always safe and effective

Most participants considered TM safe because they believed “Natural is always safe.” They said they never experienced any adverse effects of TM because of their natural origin and lack of chemicals. F11 argued: *Because these are all natural things*,* they don’t have any adverse effects.* Another participant (F5) said: *These things don’t have any adverse effects. They don’t have any chemicals*,* and chemicals are responsible for the adverse effects.* Because of a firm belief in TM’s healing power and effectiveness, most participants shared that they did not countercheck the information about TM before using it. A female participant (F15) shared that she didn’t seek information before using TM because of her beliefs. She stated: *Because these are remedies that have been used in our houses for centuries*,* we firmly believe they are correct.* It shows that participants’ information-seeking before using TM was dependent on the patient’s beliefs regarding the safety of TM. These beliefs and perceptions may be understood as indirect risks of TM modalities.

## Discussion

In this paper, we have revealed three themes: (a) *House of Knowledge*, (b) *Choosing the best possible approach for health restoration*,* and* (c) *Adverse effects of TM used*, along with six sub-themes. The participants interviewed were heterogeneous and varied significantly based on sociodemographic profiles; however, common themes developed from these interviews. The participants mentioned the influence of family in TM use and their dependence on the traditional knowledge of mothers to recover from illness. Participants also combined TM and conventional medicine practices to treat illnesses and adversities. TM was used for managing acute and chronic diseases, and modalities like herbs, food items, and supplements were commonly used. Most of the participants believed that TM modalities were safe because of their natural origin, even though some also experienced adverse effects.

### House of knowledge

This study revealed the influence of family in TM use and that mothers were the significant caregivers (and source of information) for the whole family regarding medical routines and illness. Getting medical advice from the older woman of the family is also common in other cultures [52] and aligns with other studies [1, 15, 53]. These studies highlight the differences in gender roles due to traditional and cultural norms. For instance, it was reported that the use of TM was recommended by family (usually mother) or friends [13]. Another qualitative study, including 63 participants, also revealed the role of the family in decision-making about illness [54]. In our study, some of the participants reported inquiring their mothers about preparing the herbal remedies, thus indicating the influence of family in TM use. So, despite Norway’s efficient healthcare system, seeking advice from family members about illness is transferred with the family to the new country from one generation to another, making the whole family frequently use TM.

Our findings of herbs used for adversity *(cinnamon*,* cloves*,* carom seeds*,* black cumin seeds*,* ginger*,* etc.)* are in accordance with findings among Pakistani immigrants in other European countries like Denmark and the UK [1, 15]. These herbs are also extensively used for the same medical indications in Pakistan [25, 55–58] and other countries [59]. Some herbal products (Carmina, Khamira, lal-sherbet, and hajmola) have not been reported in other studies among Pakistani immigrants in Europe before, even though they are commonly used in Pakistan. Our finding that the female participants and those with chronic illnesses used more TM modalities than the men and other participants aligns with other studies [1, 15].

### Choosing the best possible approach for health restoration

While exploring the health-seeking behavior of the participants, we found out that the majority preferred to use TM for minor illnesses. This aligns with another focus group study about the use of herbal medicines [60]. Following family traditions, positive attitudes and beliefs, avoiding the side effects of conventional medicine, and dissatisfaction with the healthcare system were the common reasons behind TM use that we identified and are following other studies [60–62]. Another important reason was the belief that TM treats the cause of illness rather than just alleviating the symptoms. It aligns with another study on Pakistani immigrants in Scotland [30]. It was also revealed that the participants managed health complaints by combining TM and conventional medicines. This behavior can be explained by the concept of medical pluralism. The concept describes how people resort to different types of medical systems for one illness due to cultural and societal influences [53]. This concept has been extensively used in studies focusing on the immigrants’ use of various types of medical practices in adversity [1, 63–65].

However, when people migrate to a place with a different culture, changes are observed in their behaviors, attitudes, values, stress, coping, and cultural identity [66, 67]. This process is called acculturation [67] and is a psychological course of adjustment to a new culture by the immigrant [68]. Participants in this study combined TM, conventional medicines, and practices learned in Norway to restore health, showing some extent of acculturation and integration. It is essential to consider that the transfer of this medical knowledge goes both ways. The participants practiced Pakistani traditions in Norway and took Norwegian medicinal knowledge and modalities (hygiene rules, food items, and medicines) to Pakistan. It can be explained by the bi-dimensional model of acculturation, which means practicing some of the previous knowledge and the new knowledge learned after the migration [67]. Other studies on Pakistani immigrants in Norway also showed some extent of acculturation regarding food habits [69], body mass index [70], and the prevalence of modern health worries [71].

### Adverse effects of TM used

Participants in the present study experienced some adverse effects (direct risk) of TM and didn’t report them to the health authorities (RELIS Manufacturer-independent drug information for healthcare professionals). Research shows that many herbal medicines from the Middle East and Asia contain various heavy metals, including lead, cadmium, mercury, etc., that can be hazardous for users [72–74]. Some medicinal plants the participants used have potentially toxic effects, e.g., psyllium husk and licorice (part of herbal supplements) [75]. Research has also shown the use of these plants among Pakistani immigrants in other parts of the world [1, 15]. It is, therefore, crucial to improve this population’s knowledge of these hazards [76].

In the herbal industry, lack of adherence to good manufacturing practices, adulteration, poor regulatory measures, and lack of quality control are common [77]. There is no worldwide consensus on how to adopt the standards for herbal drugs compared to conventional medicine [77]. Contamination of herbal medicines is a global problem [78], and research has revealed the presence of pathogenic bacteria [79] and fungal contamination [80] in various medicinal plants available in Pakistan. It may be due to poor storage, handling conditions, and transport [80]. Some herbal preparations used by the participants were bought from Pakistan. It can lead to the transfer of contaminated herbs from an unregulated market in Pakistan to Norway and can pose severe adverse effects for the users. So, research on herbal toxicology [81], standardization [82], and pharmacovigilance [83] of herbal medicines is essential and warranted.

In our study, most participants believed that TM is always safe because of its natural origin [84]. This learned behavior and robust belief system could be an indirect risk that hindered them from making good health choices. Participants showed a lack of knowledge about drug-herb interaction. This finding aligns with another qualitative study that explored women’s views on herbal medicine [85]. Another indirect risk is the lack of accurate and comprehensive labeling of herbal medicines regarding their harms and benefits [86]. Getting information about TM from the internet (social media and Google) using non-scientific sources was revealed. It aligns with another study [35] showing information variability as a common indirect risk. Usually, such information has misleading claims about effect and safety. It can be more risky for communities with low health literacy [35], which is the case with Pakistani immigrants. Without adequate skills to critically evaluate the information, there is less chance of identifying false claims about TM. A qualitative study exploring the role of evidence for patients highlighted that patients do not value scientific information about complementary medicines to the same extent as doctors do [87]. Therefore, it is a matter of concern to be considered in TM safety and risk debates.

A recent health literacy survey on immigrants in Norway revealed that they experience difficulties accessing information about disease treatment [88]. Lack of available information and access to conventional healthcare information is an indirect risk that may lead to immigrants extensively using TM. One of the reasons for using TM was the difficulty in getting medicines from their family physician. In Pakistan, medicines are used extensively for minor health complaints, and almost all conventional medications, such as antibiotics, are accessible without prescription [13]. After migration, the participants found it challenging to cope with diseases without any prescribed medicine, so they may use TM more frequently in this scenario. We emphasize that these direct and indirect risks can lead to severe consequences. It is, therefore, essential to consider them and promote measures to reduce such risks.

### Strengths and limitations

To the best of our knowledge, this is the first study that addressed the use of TM by Pakistani immigrants living in Norway. This study’s strength lies in research methods and user involvement. The use of qualitative methodology enabled us to capture the holistic view of the participants about TM [41]. User involvement helped us gain insight into the topic from the perspective of immigrants at different phases. Including members with and without medical backgrounds in the user involvement group enabled us to cover both perspectives. Open-ended questions ensured the exploration of the phenomena of interest at a deeper level. Due to information-rich participants, we obtained rich data and reached saturation after 22 interviews. Feedback from the research team on the pilot interviews helped identify the first author’s influence on the interview. She took notes during the interviews to write her views and to ensure reflexivity [48]. The detailed description of the study area, setting, participants, and the sampling and recruitment process of the participants warrant the transferability of the study findings to another setting [48]. Both the first author and the last authors read the interviews separately before starting the development of themes and agreeing on the results to ensure validity and minimize researcher subjectivity [48].

The results of this research should be interpreted considering its limitations. All the participants were interviewed once, and there was unequal distribution of participants based on gender. Future studies on the topic should consider gender balance. We asked the participants about their past experiences regarding the use of TM, so there is a possibility of recall bias. As the interviewee (SK) shared the same Pakistani culture, it may have impacted her reflexivity (the process of the researcher’s critical reflection on himself as a researcher considering assumptions, emotional reactions, expectations, and unconscious responses) despite taking appropriate measures of triangulation and feedback from the research team [48]. As qualitative research targets a specific population [40], it is essential to conduct further studies based on the type of illnesses and other parameters.

### Implications for practice and research

Knowledge from this study can help healthcare providers understand Norwegian Pakistani patients’ choices and preferences in illness. Due to the extensive use of TM by this community, it is essential to ask immigrant patients about the TM they use. Promoting research assessing the safety of medicinal plants before the effect is necessary [89]. The participants showed sparse awareness about the safety of TM and drug-herb interactions, so it is crucial to improve this community’s knowledge about this aspect. The study results show that Pakistani immigrants use TM for various types of illnesses, so there is a need to have a closer look at the TM used by this population for the diseases that are prevalent in this community. Conducting such studies on other immigrant populations in Norway is essential to gather information about their traditional practices.

## Conclusion

This study explores the experiences of TM use and perceptions of risk regarding these practices among Pakistani immigrants in Norway. The influence of family and various reasons for using TM are explored. All participants reported using TM to restore health for different diseases, alone or combined with conventional medicines. Public health strategies to improve immigrant health should consider the perceptions of immigrants regarding the use of TM. Although TM was considered safe, adverse effects were experienced and not reported to the health authorities. Further research is necessary regarding the safety and toxicity of TM, which are extensively used in Pakistani households in Norway.

## Electronic supplementary material

Below is the link to the electronic supplementary material.

Supplementary Material 1: Description. Table 4 shows the traditional and scientific names of traditional medicines, their preparation method, purpose of use, dosage, lifestyle advice, and adverse effects

Supplementary Material 2

## Data Availability

All data generated or analyzed during this study are included in this published article.

## Abbreviations

TM: Traditional Medicine
RTA: Reflexive thematic analysis
T&CM: Traditional and Complementary Medicine
WHO: World Health Organization
ADR: Adverse Drug Reactions
ID: Identification
PIB: Patient information brochure
COREQ: COnsolidated criteria for REporting Qualitative research
