# Supplementary material for: Use and perception of risk: traditional medicines of Pakistani immigrants in Norway
Source: BMC Complement Med Ther. 2024 Sep 7;24:331. doi: 10.1186/s12906-024-04620-0 (PMC11380776; doi:10.1186/s12906-024-04620-0)
Supplement: Supplementary file 2 — Supplementary Material 2 [file 12906_2024_4620_MOESM2_ESM.docx]

**Interview Guide**

1. What is the first thing you do when you experience a health complaint in Norway?
2. If you were sick in Norway, what kind of treatment did you use then? Can you share a story that happened in your life?

(Ask about Symptoms, traditional medicines used, reasons for use, perceived benefits and side effects, and sources if it didn’t come up in the story)

Ask for such instances unless the respondent has nothing else to share.

1. Do you want to mention any other information?
